# Echocardiographic chamber quantification in a healthy Dutch population

**DOI:** 10.1007/s12471-017-1035-7

**Published:** 2017-10-10

**Authors:** R. W. J. van Grootel, M. E. Menting, J. McGhie, J. W. Roos-Hesselink, A. E. van den Bosch

**Affiliations:** 1 Department of Cardiology, Erasmus MC, Rotterdam, The Netherlands; 2 Department of Radiology, Erasmus MC, Rotterdam, The Netherlands

**Keywords:** Echocardiography, 3D, Normal values, Chamber quantification

## Abstract

**Aim:**

For accurate interpretation of echocardiographic measurements normative data are required, which are provided by guidelines. For this article, the hypothesis was that these cannot be extrapolated to the Dutch population, since in Dutch clinical practice often higher values are found, which may not be pathological but physiological. Therefore this study aimed to 1) obtain and propose normative values for cardiac chamber quantification in a healthy Dutch population and 2) determine influences of baseline characteristics on these measurements.

**Methods:**

Prospectively recruited healthy subjects, aged 20–72 years (at least 28 subjects per age decade, equally distributed for gender) underwent physical examination and 2D and 3D echocardiography. Both ventricles and atria were assessed and volumes were calculated.

**Results:**

147 subjects were included (age 44 ± 14 years, 50% female). Overall, feasibility was good for both linear and volumetric measurements. Linear and volumetric parameters were consistently higher than current guidelines recommend, while functional parameters were in line with the guidelines. This was more so in the older population. 3D volumes were higher than 2D volumes. Gender dependency was seen in all body surface area (BSA) corrected volumes and with increasing age, ejection fractions decreased.

**Conclusion:**

This study provides 2D and 3D echocardiographic reference ranges for both ventricles and atria derived from a healthy Dutch population. BSA indexed volumes are gender-dependent, age did not influence ventricular volumes and a rise in blood pressure was independently associated with increased right ventricular volumes. The higher volumes found may be indicative for the Dutch population being the tallest in the world.

## Introduction

Echocardiography is indispensable in clinical practice. It is the most widely used non-invasive imaging tool to assess and quantify cardiac structural and functional parameters, mainly because of its versatility: it is widely available, relatively cost-effective and mobile. To interpret the performed measurements, solid normative data are essential. The recently revised guideline for cardiac chamber quantification provides guidance for echocardiographic assessment [1, 2]. For some measurements, age- and gender-specific values are reported.

In the literature, there are four studies that show an influence of gender and age on left ventricular (LV) and right ventricular (RV) volumes [3]. However, besides age and gender, there are implications that ethnicity also influences cardiac size and function [4–7]. Echocardiographic measurements performed in healthy Dutch people, the tallest people worldwide [8], often supersede the upper limits of normal (ULN) given by current guidelines though most indices are corrected with body surface area (BSA). This implies that BSA correction does not fully equalise differences originating from height. Also, a study comparing Caucasians with Asian Indians showed smaller 3D LV volumes in Asians but with a comparable ejection fraction (EF). However, ethnic-specific normal values for the Dutch population are not yet available.

Therefore, this study aims to provide 1) age-specific reference values for echocardiographic chamber measurements specifically in a Dutch population and 2) identify the influence of age, gender, body mass index (BMI), BSA and blood pressure on the echocardiographic measurements.

## Methods

### Patient population

Healthy volunteers, aged 20–72 years, were enrolled in 2014–2015 for this prospective cross-sectional study and were stratified into five groups: 20–29, 30–39, 40–49, 50–59 and 60–72 years (each 50% female). Details have been published earlier [9]. Healthy subjects were examined at the cardiology outpatient clinic of the Erasmus Medical Centre, Rotterdam. Exclusion criteria were: (prior) cardiovascular disease, systemic disease, or cardiac medication, the finding of cardiac abnormalities during examination or cardiovascular risk factors consisting of hypertension (systolic and diastolic blood pressure >140/80 mm Hg), diabetes mellitus and hypercholesterolaemia. Professional athletes, morbidly obese subjects (BMI >40 kg/m^2^), pregnant women and women with breast implants were excluded. This study was carried out according to the principles of the Declaration of Helsinki and approved by the local ethics committee. Written informed consent was obtained from all patients.

### Clinical assessment

The study consisted of a questionnaire on medical history and health status, physical examination, venous blood sampling, 12-lead electrocardiogram and an echocardiogram.

### Echocardiographic image acquisition

Echocardiographic studies were carried out by one of two experienced sonographers. Two-dimensional greyscale harmonic images were obtained in the left lateral decubitus position using an i33 or EPIQ7 ultrasound system (Philips Medical Systems, Best, the Netherlands) equipped with a transthoracic broadband X5-1 matrix transducer (composed of 3,040 elements with 1–5 MHz). Dedicated views were taken for assessment of both atria and ventricles. The studies were stored in Digital Imaging and Communications in Medicine (DICOM) format.

### Echocardiographic measurements

The current recommendations for chamber quantification were used [1]. LV end-diastolic and -systolic diameters were measured in the parasternal short axis view, together with their respective volumes using the apical four- and two-chamber views. Volumes were measured using the method-of-disk summation technique.

The left atrial (LA) area was measured in dedicated atrial four- and two-chamber views to avoid foreshortening. Biplane volumes were measured using the method-of-disk summation technique.

For the right ventricle, end-diastolic and -systolic areas (EDA and ESA) were measured in order to calculate the fractional area change:

1 $$FAC=\frac{enddiastolicarea-endsystolicarea}{enddiastolicarea}*100)$$

**Fig. 1:**
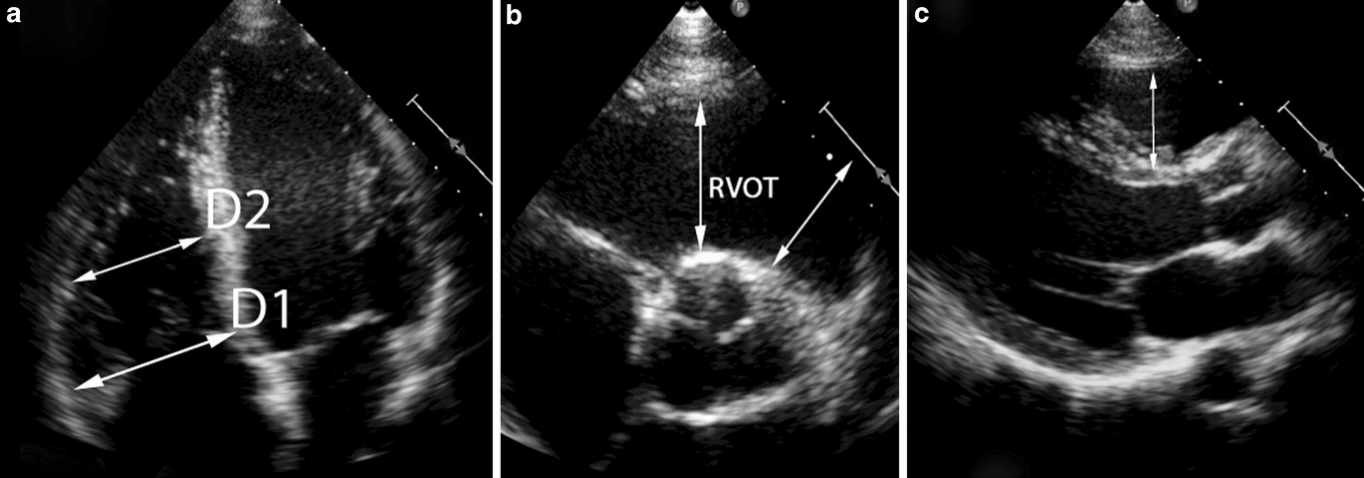
Measurements of RV dimensions. The RV basal (D1) and mid cavity (D2) dimensions are shown in the RV focused apical four-chamber view (**a**). Measurements of the right ventricular outflow tract at the proximal level and the distal level are shown in the parasternal short-axis (**b**) and parasternal long-axis view (**c**)

End-diastolic basal- and mid-cavity diameters were measured in the modified apical four-chamber view. The proximal and distal outflow tract diameters were measured in the parasternal short-axis view (Fig. 1). Tricuspid annular plane systolic excursion (TAPSE) was measured.

For the right atrium, the single plane volume was measured in the apical four-chamber view using the method-of-disk summation technique and the area length method.

### Three-dimensional measurements

LV volumetric measurements were performed with TomTec 4D LV function suite (4D LV Analysis; TomTec Imaging Systems, Unterschleissheim, Germany) using a semi-automated endocardial border trace, after the apex and the middle of the mitral valve annulus are determined by the operator. The end systolic and diastolic frame are automatically detected in order to start contour detection. Corrections to the endocardial trace were manually applied by the operator if needed. For the right ventricle, a similar routine was used (TomTec 4D RV function; TomTec Imaging Systems, Unterschleissheim, Germany).

### Statistical analysis

Normal distribution was checked using Shapiro-Wilks tests and histograms. Depending on data distribution, continuous data are presented as mean ± standard deviation (SD) or median with first-third quartile (Q1-Q3). Categorical data are presented as frequencies and percentages. Student’s t‑test, the Mann-Whitney-U test, χ^2^-test or Fisher’s exact test was used when appropriate. Correlations were measured using the Pearson correlation test. For correlations between echocardiographic parameters and age, linear regression analysis was applied. Variables that reached significance and did not show collinearity with other variables were included in a multivariable model. When collinearity did occur, the variable with the highest correlation coefficient was included. A *p* ≤ 0.05 was considered statistically significant. Statistical analysis was done using the Statistical Package for Social Science version 21 (IBM DPDD Statistics for Windows, Armonk, New York, USA).

## Results

**Fig. 2:**
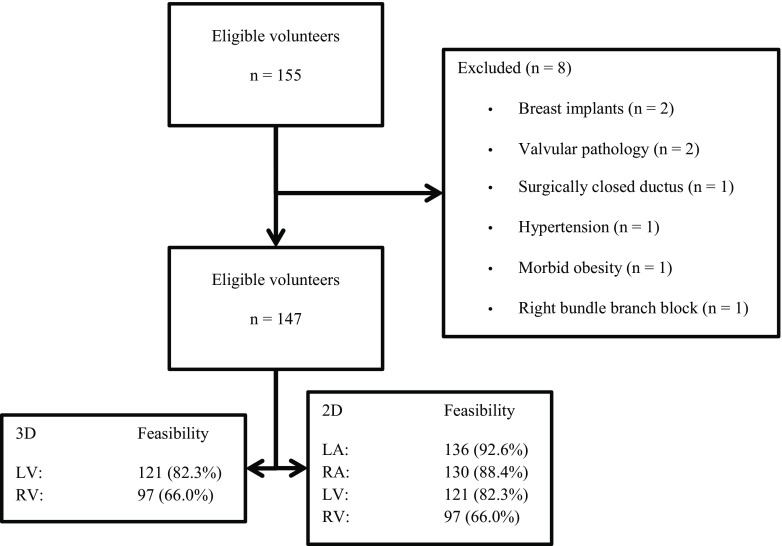
Flowchart depicting the inclusion and feasibility of the performed measurements

Of the 155 subjects eligible, 147 were included (mean age 44.6 ± 13.7 years, 50% female) (Fig. 2). Tab. 1 shows the characteristics of the study population. LV volume and function assessment with 3D echocardiography was feasible in 121 (82.3%) volunteers; RV volume and function assessment with 3D echocardiography was feasible in 97 (66.0%) volunteers. Height, weight, BMI, BSA and systolic and diastolic blood pressure were significantly lower in women.

**Table 1:** Baseline characteristics

	Total population	Male	Female
	*n* = 147	*n* = 73	*n* = 74
Age (years)	44.6 ± 13.8	44.0 ± 13.7	45.3 ± 13.8
Height (cm)	175 ± 9	181 ± 7	169 ± 6
Weight (kg)	75 ± 13	82 ± 11	67 ± 9
Body mass index (kg/m^2^)	24.4 ± 3.3	25.2 ± 3.3	23.6 ± 3.0
Body surface area (m^2^)	1.89 ± 0.19	2.03 ± 0.15	1.76 ± 0.12
Systolic blood pressure (mm Hg)	127 ± 15	131 ± 16	123 ± 12
Diastolic blood pressure (mm Hg)	80 ± 9	82 ± 9	77 ± 9
Heart rate (beats per minute)	71 ± 12	69 ± 12	72 ± 12

### Echocardiographic chamber measurements in relation with age

Tab. 2 presents an overview of echocardiographic parameters per age decade. For 3D derived volumes, the averages for the left ventricle were: end-diastolic volume (EDV) 78 ± 12 ml/m^2^ and end-systolic volume (ESV) 34 ± 6 ml/m^2^. For the right ventricle, values were 58 ± 12 ml/m^2^ and 24 ± 6 ml/m^2^. LV and RV volumes were not age-dependent; this was true for both 2D and 3D echocardiography. Age was inversely correlated with LVEF (57 ± 4 ml/m^2^ in the youngest group and 55 ± 3 ml.m^2^ in the oldest group (*r*: −0.229, *p*: 0.012)). RVEF was 58 ± 4 ml/m^2^ and was not correlated with age. Importantly, volumetric measurements acquired with 2D echocardiography all exceeded the ULN as recommended in the guideline. This was also true for 3D LV volumes, but not for the 3D RV volumes.

**Table 2:** Echocardiographic variables per age decade

	Total population	20–29 years	30–39 years	40–49 years	50–59 years	60–72 years		
	*n* = 147	*n* = 32	*n* = 28	*n* = 28	*n* = 31	*n* = 28	R	*p*-value
**2D measurements**								
*Left ventricle*								
EDV (ml/m^2^)	62.4 ± 10.1	63.9 ± 9.5	63.1 ± 9.9	64.1 ± 11.0	60.5 ± 8.0	60.6 ± 12.2	ns	0.061
ESV (ml/m^2^)	24.9 ± 5.8	25.4 ± 5.1	24.7 ± 6.2	26.3 ± 5.5	23.2 ± 5.1	25.0 ± 6.9	ns	0.349
EF (%)	60.4 ± 4.7	60.4 ± 3.6	61.4 ± 5.0	59.2 ± 4.5	61.9 ± 5.3	59.2 ± 4.5	ns	0.596
EDD (mm)	46 ± 4	46 ± 3	47 ± 3	45 ± 4	45 ± 5	44 ± 5	**0.248**	**0.003**
ESD (mm)	28 ± 4	29 ± 3	28 ± 3	28 ± 5	28 ± 4	28 ± 6	ns	0.566
*Right ventricle*								
EDA (cm^2^/m^2^)	13.2 ± 2.3	14.2 ± 2.3	13.5 ± 2.5	12.9 ± 1.8	12.3 ± 2.0	12.6 ± 2.4	**0.275**	**0.001**
ESA (cm^2^/m^2^)	7.7 ± 1.7	8.3 ± 1.6	7.7 ± 1.7	7.4 ± 1.6	7.3 ± 1.7	7.2 ± 2.0	**0.219**	**0.014**
FAC (%)	42.8 ± 7.3	41.2 ± 6.6	43.6 ± 6.2	42.6 ± 8.5	42.6 ± 8.7	44.5 ± 7.2	ns	0.198
D1 (mm)	39 ± 5	39 ± 5	38 ± 5	39 ± 6	38 ± 4	40 ± 6	ns	0.804
D2 (mm)	29 ± 5	30 ± 6	28 ± 4	31 ± 5	28 ± 5	30 ± 6	ns	0.795
RVOT proximal PLAX (mm)	32 ± 4	30 ± 4	30 ± 4	31 ± 4	33 ± 5	34 ± 4	**0.355**	**<0.001**
RVOT proximal SAX (mm)	31 ± 4	31 ± 4	30 ± 4	32 ± 4	32 ± 5	31 ± 3	**0.174**	**0.047**
RVOT distal SAX (mm)	22 ± 3	22 ± 2	23 ± 2	21 ± 3	23 ± 2	23 ± 3	ns	0.326
Longitudinal diameter (mm)	82 ± 8	84 ± 7	86 ± 8	81 ± 8	81 ± 7	79 ± 7	**0.181**	**0.029**
TAPSE (mm)	26 ± 4	26 ± 4	27 ± 3	25 ± 4	26 ± 3	26 ± 4	ns	0.267
*Left atrium*								
Length A4C (mm)	5.0 ± 0.6	4.9 ± 0.6	5.0 ± 0.6	5.0 ± 0.5	5.1 ± 0.5	5.2 ± 0.7	ns	0.064
Area A4C (cm^2^)	17.3 ± 3.2	16.6 ± 2.4	17.2 ± 3.0	17.1 ± 3.8	17.8 ± 2.4	17.8 ± 4.0	ns	0.134
Max volume (ml/m^2^)	28.8 ± 7.2	27.8 ± 5.7	28.1 ± 6.6	29.0 ± 9.2	29.4 ± 5.5	30.0 ± 9.1	ns	0.170
*Right atrium*								
Length (cm/m^2^)	2.6 ± 0.3	2.6 ± 0.3	2.6 ± 0.3	2.5 ± 0.3	2.6 ± 0.3	2.8 ± 0.3	ns	0.089
Area (cm/m^2^)	8.9 ± 1.5	8.8 ± 1.3	9.3 ± 1.5	8.5 ± 1.4	8.6 ± 1.7	9.2 ± 1.2	ns	0.866
Max volume (ml/m^2^)	24.5 ± 7.0	24.4 ± 6.0	26.7 ± 8.4	23.3 ± 6.5	23.2 ± 7.9	24.8 ± 5.7	ns	0.343
**3D measurements**								
*Left ventricle*								
EDV (ml/m^2^)	77.5 ± 12.3	81.2 ± 12.1	77.1 ± 8.9	77.2 ± 10.7	75.6 ± 8.9	75.1 ± 18.9	ns	0.054
ESV (ml/m^2^)	33.9 ± 6.2	34.9 ± 6.2	32.9 ± 4.9	34.0 ± 5.2	33.4 ± 5.8	33.9 ± 8.8	ns	0.603
EF (%)	56.3 ± 3.7	57.0 ± 3.6	57.4 ± 3.1	55.9 ± 4.1	56.0 ± 4.3	54.8 ± 2.9	**−0.229**	**0.012**
*Right ventricle*								
EDV (ml/m^2^)	57.7 ± 11.9	58.2 ± 14.3	54.9 ± 7.0	56.4 ± 12.1	58.5 ± 10.2	61.5 ± 15.0	ns	0.403
ESV (ml/m^2^)	24.3 ± 5.8	23.7 ± 6.8	22.7 ± 4.4	24.2 ± 6.0	24.3 ± 4.8	27.5 ± 6.5	ns	0.054
EF (%)	58.0 ± 4.2	59.1 ± 3.7	59.0 ± 4.3	57.3 ± 4.7	58.7 ± 4.1	54.9 ± 3.1	**−0.298**	**0.003**

*EDV* end-diastolic volume, *ESV* end-systolic volume, *EF* ejection fraction, *EDD* end-diastolic diameter, *ESD* end-systolic diameter, *EDA* end-diastolic area, *ESA* end-systolic area, *FAC* fractional area change, *RVOT* right ventricular outflow tract, *SAX* short-axis view, *TAPSE* tricuspid annular plane systolic excursion.

### Echocardiographic chamber measurements in relation with gender

**Fig. 3:**
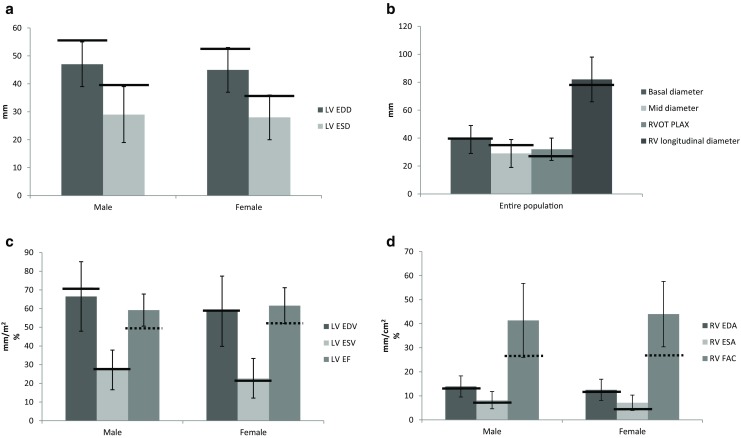
Four charts depicting 2D measurements, the error bars are set at 2 SD. The solid and dotted lines represent the upper and lower limits of normal as stated in the guideline. Left ventricular systolic and diastolic diameters per gender (**a**). Right ventricular linear measurements are presented (**b**). Left ventricular 2D volumes are given per gender (**c**) as well as the right ventricular areas and fractional area change (**d**)

Tab. 3 shows echocardiographic chamber measurements per gender. Fig. 3 shows 2D measurements for both ventricles (error bars at 2SD), with bars depicting the ULN (solid line) or lower limit (dotted line) of normal according to the guidelines [1]. Most of the measured variables showed gender-dependency. 2D and 3D LV and RV dimensions and volumes were gender-dependent. 2D and 3D EF was also gender-dependent with the exception of 3D RVEF. After BSA indexation, males had higher ventricular volumes. BSA indexation of LA volume negated gender differences, but BSA indexed right atrial (RA) volumes remained bigger in men than women. On average, LA maximum volume was 28.8 ± 7.2 ml/m^2^, with 25% of the study population having LA dilatation (>34 ml/m^2^) according to the guideline. In this study, the ULN was 43.2 ml/m^2^.

**Table 3:** Echocardiographic variables per gender

	Total population	Male	Female	
	*n* = 147	*n* = 73	*n* = 74	*p*-value
**2D measurements**				
*Left ventricle*				
2D EDV (ml/m^2^)	62.4 ± 10.1	66.5 ± 9.3	58.6 ± 9.4	**<0.001**
2D ESV (ml/m^2^)	24.9 ± 5.8	27.2 ± 5.3	22.7 ± 5.3	**<0.001**
2D EF (%)	60.4 ± 4.7	59.2 ± 4.3	61.6 ± 4.8	**0.002**
EDD (mm)	46 ± 4	47 ± 4	45 ± 4	**0.002**
ESD (mm)	28 ± 4	29 ± 5	28 ± 4	**0.026**
*Right ventricle*				
EDA (cm^2^/m^2^)	13.2 ± 2.3	13.9 ± 2.2	12.5 ± 2.2	**<0.001**
ESA (cm^2^/m^2^)	7.7 ± 1.7	8.2 ± 1.8	7.1 ± 1.6	**<0.001**
FAC (%)	42.8 ± 7.3	41.4 ± 7.7	44.0 ± 6.8	**0.048**
D1 (mm)	39 ± 5	41 ± 5	37 ± 4	**<0.001**
D2 (mm)	29 ± 5	32 ± 5	27 ± 5	**<0.001**
RVOT proximal PLAX (mm)	32 ± 4	33 ± 5	31 ± 4	**0.037**
RVOT proximal SAX (mm)	31 ± 4	32 ± 4	31 ± 4	**0.013**
RVOT distal SAX (mm)	22 ± 3	23 ± 3	22 ± 3	0.15
Longitudinal diameter (mm)	82 ± 8	85 ± 7	80 ± 7	**0.014**
TAPSE (mm)	26 ± 4	26 ± 4	26 ± 4	0.344
*Left atrium*				
Length A4C (mm)	5.0 ± 0.6	5.2 ± 0.6	4.9 ± 0.6	**0.019**
Area A4C (cm^2^)	17.3 ± 3.2	17.8 ± 3.4	16.9 ± 2.8	0.086
Biplane max volume (ml/m^2^)	28.8 ± 7.2	27.9 ± 7.5	29.8 ± 6.9	0.135
*Right atrium*				
Length (cm/m^2^)	2.6 ± 0.3	2.5 ± 0.3	2.7 ± 0.3	**<0.001**
Area (cm/m^2^)	8.9 ± 1.5	8.9 ± 1.6	8.8 ± 1.3	0.626
Single plane MOD max volume (ml/m^2^)	24.5 ± 7.0	25.8 ± 8.1	23.3 ± 5.7	**0.038**
**3D measurements**				
*Left ventricle*				
3D EDV (ml/m^2^)	77.5 ± 12.3	80.7 ± 13.6	74.2 ± 9.9	**0.003**
3D ESV (ml/m^2^)	33.9 ± 6.2	35.9 ± 6.7	31.8 ± 5.0	**<0.001**
3D EF (%)	56.3 ± 3.7	55.5 ± 3.3	57.1 ± 3.9	**0.017**
*Right ventricle*				
3D EDV (ml/m^2^)	57.7 ± 11.9	61.3 ± 12.3	54.0 ± 10.4	**0.002**
3D ESV (ml/m^2^)	24.3 ± 5.8	26.0 ± 6.2	22.5 ± 4.9	**0.003**
3D EF (%)	58.0 ± 4.2	57.5 ± 4.1	58.4 ± 4.3	0.315

*EDV* end-diastolic volume, *EF* ejection fraction, *EDD* end-diastolic diameter, *ESD* end-systolic diameter, *EDA* end-diastolic area, *ESA* end-systolic area, *FAC* fractional area change, *RVOT* right ventricular outflow tract, *PLAX* parasternal long-axis, *SAX* short-axis, *TAPSE* tricuspid annular plane systolic excursion

**Fig. 4:**
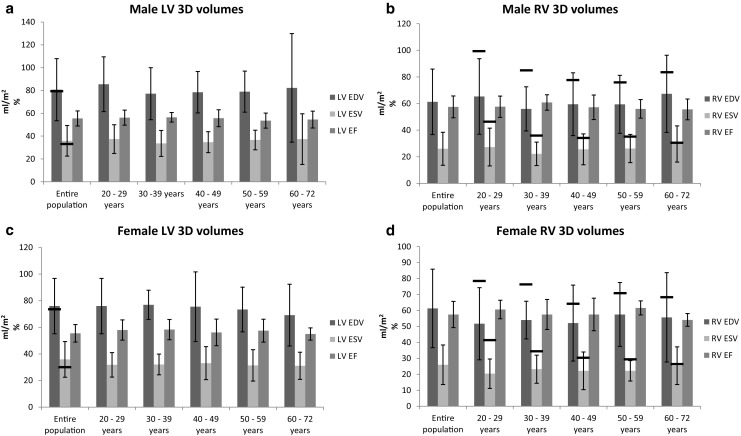
Four charts showing 2D and 3D derived left and right ventricular volumes and ejection fractions, for the entire study population and per age group. The error bars are set at 2SD, and the solid lines represent the upper limits of normal according to the guideline

Fig. 4 shows age- and gender-specific volumes for both the LV and RV 3D data and the ULN according to the guideline are again depicted. EF for both ventricles was significantly correlated with age, EDV and ESV did not correlate.

### Correlations

BSA correlated strongest with echocardiographic parameters compared with height, weight or BMI. Ventricular volumes were not correlated with age, and LVEF and RVEF showed a decrease with age. RV volumes showed a linear increase with higher blood pressure and heart rate, also after correcting for heart rate, QRS duration and gender.

Agreement between 2D and 3D for the LV measurements was moderate: LV EDV: *r*: 0.643, *p* < 0.001, and LV ESV *r*: 0.583, *p* < 0.001, respectively. For the RV correlations between 2D volumes and 3D areas were weak: 3D EDV vs 2D EDA *r*: 0.295, *p*: 0.004 and ESV *r*: 0.295, *p*: 0.005 respectively.

## Discussion

This prospective study presents normative data for echocardiographic chamber quantification, age- and gender-specific, in a healthy Dutch population. Overall, linear and volumetric parameters exceeded the ULN as stated in the guideline, while functional parameters agreed with them. BSA remains the best variable for indexation.

### Feasibility

Feasibility of LV measurements in this study is in line with previous studies [4, 10, 11]. Feasibility is important when a technique is considered for clinical use and the percentages in this study are good, especially since subjects with a poor acoustic window were not excluded. The need for implementation of 3D echocardiography for LV volume and EF in clinical use is high. The lower feasibility for the right ventricle was expected given its location in the thorax right behind the sternum. Higher percentages have been reported in the literature but poor acoustic windows were an exclusion criterion whereas this was not the case for this study [3].

### Differences with guidelines

Comparing the results from this study to the ULN as stated in the guideline[1], we found some discrepancies. For most of the parameters, ULN of this study exceeded those of the guideline. This was true for linear and volumetric measurements, regardless of which ventricle or atrium the parameter belonged to: both were larger, this is especially outspoken in the elderly. For instance, when looking at 2D LV volumes: the older the group, the larger the difference. The higher linear and volumetric parameters found may be indicative to the Dutch people being the tallest people in the world, which stresses the importance of specific normative echocardiographic data for the Dutch population. Comparing our data with results from the NORRE trial, a European multicentre study pertaining to primarily white individuals, we found that our volumes and dimensions were generally higher [12]. Mean BSA in their study was 1.8 ± 0.2 vs our 1.89 ± 0.19 m^2^. This was due to both a higher weight (65 ± 12 vs 75 ± 13 kg) and height (169.8 ± 9.6 vs 175 ± 9 cm), meaning that these higher values are not due to leaner subjects.

Given that the Dutch population is the tallest worldwide [8], the question arose whether BSA was the best parameter for indexation. Univariate analysis in this study showed that BSA correlated strongest when compared with height, weight or BMI; it was indeed the best candidate for indexation. Most parameters exceeded the guideline, with one exception: 3D derived volumes of the right ventricle were in line with the guideline and current literature [3]. Functional parameters concerning LV and RV systolic function (EF, RV FAC and TAPSE) agreed with the proposed values in the guideline, meaning that the current guideline is applicable for the Dutch population.

Multiple studies reported on the negative relation between age and echocardiographic LV volumes[10, 11, 13], which is similar to what is seen in CMR studies [14, 15]. In this study, LV volumes did not decrease with age while LVEF did. For the RV, there was no positive linear correlation in RVEF as is reported by Maffessanti et al. (*r* 0.240, *p* < 0.01) [3] , but a negative correlation. This probably reached significance because of the lower mean RVEF in the oldest group. Between the age groups 20–29 to 50–59 years, values remain practically the same. Indeed, the correlation between age and RVEF became stronger when the study population was divided into groups ≤55 years and >55 years. Maffessanti et al. reported similar values, but in their cohort the oldest group (age >70 years) reported higher values for RVEF. Perhaps RVEF does not increase until old age (>70 years), which might explain why our results are different. Clearly, the influence of age needs further exploration.

### Correlations with blood pressure

Hypertension was a reason for exclusion, but it is worth mentioning that some volunteers had a higher blood pressure but when checked again by a general practitioner were found to be normal. These volunteers were not excluded. Analysis showed that higher blood pressure led to higher RV volumes. This could imply that sufficient blood pressure regulation could influence the right ventricle.

### Clinical implications

The reference values in this study could lead to some changes in clinical decision-making. Though most guidelines use functional parameters, there are situations where volumes or linear dimensions are being used. For instance, when diagnosing LV diastolic dysfunction, LA dilatation plays an important role [16]. With that in mind, in patients suspected of heart failure with mid-range EF, LA enlargement is used to substantiate the diagnosis [17]. An enlarged left atrium is also a strong prognosticator in, for instance, in the recurrence of atrial fibrillation after surgery [18].

RV size is of particular importance for patients with adult congenital heart disease. In patients with an atrial septal defect, if the right ventricle is enlarged intervention is warranted; this is also true for patients with ventricular septal defect [19].

The impact is smaller regarding valvular disease; LV linear dimensions are still used as opposed to volumes [20], and values of LV linear dimensions in this study did agree with guidelines.

### Limitations

Considering the size and Dutch ethnicity of the study population, conclusions drawn should be interpreted with caution.

## Conclusion

This study presents age- and gender-specific normative data specifically for the native Dutch population, and reveals that linear and volumetric parameters exceed the ULN while functional parameters agree with the guideline. The higher linear and volumetric parameters found may be indicative to native Dutch people being the tallest people worldwide. The authors suggest specific normative values for echocardiographic assessment in the Dutch population.
